# Nitrogen addition determines whether invasive plant-derived biochar facilitates or suppresses *Flaveria bidentis* invasion

**DOI:** 10.3389/fpls.2026.1942632

**Published:** 2026-10-28

**Authors:** Xin Pu, Huijun Wu, Jin Zheng, Mengxuan He, Jie Liu

**Affiliations:** 1 State Key Laboratory of Petroleum Pollution Control, China National Petroleum Corporation Research Institute of Safety & Environment Technology, Beijing, China; 2 State Key Laboratory of Herbage Improvement and Grassland Agro-ecosystems, Center for Grassland Microbiome, College of Pastoral Agriculture Science and Technology, Lanzhou University, Lanzhou, China; 3 Faculty of Geography, Tianjin Normal University, Tianjin, China

**Keywords:** coastal saline–alkaline wetlands, community invasion resistance, *Flaveria bidentis* invasion, invasive plant-derived biochar, nitrogen enrichment

## Abstract

Invasive plant-derived biochar offers a promising strategy for converting invasive biomass into valuable soil amendments. However, its effects on plant invasion under increasing nitrogen deposition remain uncertain. Here, we established coastal saline–alkaline wetland microcosms containing native plant communities invaded by *Flaveria bidentis.* We evaluated biochars derived from two invasive plants, *Amaranthus palmeri* (AP) and *Spartina alterniflora* (SA), applied at 1%, 3%, and 5% (w/w), with or without nitrogen addition. We quantified invasive plant performance, native community responses, invasibility, invasion resistance, niche characteristics, and phylogenetic structure. Biochar effects were strongly dependent on feedstock, application rate, and nitrogen availability. In the absence of nitrogen addition, applying AP-biochar or SA-biochar at either 3% or 5% increased the relative invasion risk by enhancing the performance of *Flaveria bidentis* and weakening the resistance of the native community. Nitrogen addition altered these outcomes but did not consistently suppress relative invasion. AP-biochar with nitrogen generally reduced invasive plant dominance and enhanced community resistance. The 3% AP-biochar + nitrogen most effectively suppressed *F. bidentis* growth, whereas 5% AP-biochar + nitrogen maximized invasion resistance and diversity recovery. In contrast, 1% and 3% SA-biochar + nitrogen frequently promoted *Flaveria bidentis*, while 5% SA-biochar showed limited suppression. These findings reveal that the ecological effects of invasive plant-derived biochar depend on feedstock, application rate, and nitrogen availability. Careful feedstock selection and rate optimization are therefore essential for developing biochar-based strategies to restore invasion-resistant coastal wetland ecosystems.

## Highlights

- Invasive plant-derived biochar can promote *Flaveria bidentis* invasion at specific doses.
- Biochar feedstock identity determines invasion risk and restoration outcomes.
- AP-biochar + nitrogen suppressed *F. bidentis* and enhanced community resistance.
- Low-dose SA-biochar plus nitrogen stimulated invader growth despite diversity gains.

## 1 Introduction

Plant invasions have become one of the leading drivers of global biodiversity loss and ecosystem degradation owing to the exceptional environmental adaptability, rapid reproduction, and efficient dispersal of invasive species (Jiao et al., 2023; Khatri et al., 2024a). Following establishment, invasive plants can profoundly alter community composition, disrupt ecosystem functioning, reduce biodiversity, and even threaten human health through ecological and socioeconomic impacts (Jiao et al., 2023; Kamel et al., 2025). Consequently, developing effective, economically viable, and environmentally sustainable strategies for invasive plant management has become an urgent priority worldwide (Khatri et al., 2024b; Lele et al., 2025). Existing management approaches, including mechanical removal, herbicide application, biological control, and utilization-based management, primarily focus on suppressing invasive populations (Swierszcz et al., 2024; Chambers et al., 2025; Lele et al., 2025). However, conventional control methods are frequently constrained by high labor and economic costs, incomplete eradication, and recurrent reinvasion (Swierszcz et al., 2024; Xu et al., 2026). Consequently, increasing attention has been directed toward converting invasive plant biomass into value-added products, thereby integrating invasive species control with sustainable biomass utilization and circular bioeconomy strategies (Feng et al., 2021).

Among these utilization pathways, biochar has attracted considerable interest because of its broad applications in sustainable agriculture, ecosystem restoration, environmental remediation, and carbon sequestration (Bitarafan et al., 2019; Jia et al., 2026). Nevertheless, the relatively high production cost of biochar remains a major obstacle to its widespread implementation (Feng et al., 2021). Invasive plants, characterized by abundant biomass, rapid regeneration, wide distribution, and low harvesting costs, represent attractive alternative feedstock for biochar production (Khatri et al., 2024a). Compared with conventional agricultural residues, invasive plant biomass may therefore provide distinct economic and logistical advantages for large-scale biochar production (Khatri et al., 2024b; Yin et al., 2024). However, recycling invasive plants into biochar may also generate unintended ecological consequences that warrant careful evaluation before practical application (Fartyal et al., 2025; Li et al., 2025d). Biochar can directly influence plant growth while indirectly regulating invasion processes through modifications of soil physicochemical properties, nutrient availability, microbial communities, and plant–soil feedbacks, thereby reshaping competitive interactions between invasive and native species (Yin et al., 2024). Previous studies have demonstrated that invasive plant-derived biochar can alter plant biomass, species diversity, and community composition (Bieser et al., 2022; Fartyal et al., 2025). However, reported effects of heterospecific invasive plant-derived biochar have been inconsistent, ranging from suppression to facilitation of invasive plants depending on biochar feedstock, soil environmental conditions, and plant species identity (Yin et al., 2024). Consequently, whether heterospecific invasive biochar ultimately enhances or weakens invasion success, and whether these effects arise through stimulation of invasive plant performance, suppression of native communities, or simultaneous effects on both, remains unresolved.

The ecological consequences of invasive plant-derived biochar are likely to become increasingly important in the context of global environmental change. Coastal saline–alkaline wetlands are among the ecosystems most vulnerable to biological invasion because their plant communities are frequently exposed to multiple anthropogenic disturbances, particularly increasing atmospheric nitrogen (N) deposition (Xu et al., 2026). Nitrogen enrichment generally increases resource availability, relaxes nutrient limitation, and preferentially benefits fast-growing invasive species, thereby enhancing ecosystem invasibility and reducing the biotic resistance of native communities (Li et al., 2025a; Meng et al., 2026b). Simultaneously, biochar can modify nutrient retention, soil pH, water availability, and microbial activity, suggesting that its ecological effects may strongly depend on ambient nutrient conditions (Meng et al., 2026b; Tang et al., 2026). Consequently, nitrogen enrichment may either amplify or offset biochar-mediated changes in plant performance and competitive interactions through complex, non-additive effects on resource acquisition and community assembly. Despite increasing recognition of the individual effects of biochar and nitrogen deposition, their interactive influences on invasive plant performance, native community resistance, and invasion dynamics remain poorly understood, particularly in saline–alkaline coastal wetlands where nitrogen deposition is rapidly intensifying (He et al., 2026; Meng et al., 2026a).

Importantly, previous studies have predominantly evaluated invasive plant-derived biochar by examining changes in soil properties or individual plant growth, whereas considerably less attention has been devoted to the ecological mechanisms governing plant community assembly during invasion (Yang et al., 2022). Plant invasion is fundamentally a community assembly process driven by shifts in functional trait expression, species interactions, competitive hierarchies, and niche differentiation (Li et al., 2026). Therefore, assessing only biomass responses provides limited insight into the mechanisms underlying invasion success or community resistance. Integrating multiple community-level indicators, including plant functional traits, species diversity, invasion susceptibility, community resistance, competitive advantage, relative invasiveness, and niche breadth and overlap, can provide a more comprehensive understanding of how invasive plant-derived biochar reshapes competitive interactions and determines invasion outcomes (Yan et al., 2023). Such a mechanistic perspective is essential for evaluating not only the ecological risks associated with recycling invasive biomass into biochar but also its potential as a sustainable management strategy under ongoing global change.

To address these knowledge gaps, we established experimental saline–alkaline wetland plant communities consisting of native species invaded by *Flaveria bidentis* and evaluated the effects of biochars derived from two heterospecific invasive plant species (*Amaranthus palmeri* and *Spartina alterniflora*) under ambient and nitrogen-enriched conditions. By simultaneously quantifying plant functional traits, community diversity, invasion susceptibility, community resistance, competitive advantage, relative invasiveness, and niche breadth and overlap, we sought to elucidate the mechanisms through which heterospecific invasive plant-derived biochar and nitrogen enrichment regulate invasion dynamics and plant community assembly. Specifically, we hypothesized that (1) heterospecific invasive plant-derived biochar would enhance the competitive performance and relative invasiveness of *F. bidentis* while suppressing native plant growth and reducing community resistance; and (2) nitrogen enrichment would interact with heterospecific invasive biochar by altering competitive interactions, thereby regulating plant diversity, niche differentiation, invasion intensity, and overall community stability.

## 2 Materials and methods

### 2.1 Soil collection and biochar preparation

In September 2021, soil was collected from a 600 m×100 m coastal saline–alkaline wetland in Tianjin, China. Ten sampling plots were established, and eight 1 m × 1 m quadrats were randomly selected within each plot. Surface litter, dead plant material, and stones were removed before sampling. Surface soil (0–20 cm) was collected from each quadrat and composited at the plot level. The ten composite samples were then thoroughly mixed and homogenized to minimize spatial variation in soil properties and soil seed-bank composition. The homogenized soil was passed through a 2-mm sieve after removal of stones and coarse roots and was subsequently used to establish the experimental plant communities. The geographic coordinates are provided in **Supplementary Table S1**. The experimental soil had a salinity of 0.84 g kg^−1^ and a pH of 8.5 (**Supplementary Table S2**).

The sampling area was characterized by clay-rich, low-porosity saline–alkaline soil and was dominated by the native species *Suaeda glauca*, *Chenopodium album*, *Setaria viridis*, and *Kochia scoparia*, together with the invasive species *F. bidentis*. The region has a warm temperate monsoon climate, with a mean annual temperature of 12.3°C and annual precipitation of approximately 566 mm. Importantly, although *A. palmeri* and *S. alterniflora* occurred within the broader study area, soil for the greenhouse experiment was collected specifically from designated sampling plots that were not occupied by these two species. Thus, the soil seed bank used to establish the experimental communities contained *F. bidentis* and native plant species but did not contain seeds of the two invasive species used as biochar feedstocks.

Biochars were produced from the aboveground biomass of *A. palmeri* and *S. alterniflora*, collected separately from the same soil sampling area. The plant materials were washed, oven-dried at 72°C for 36 h, and pyrolyzed at 550°C. The resulting biochars were ground, passed through a 2-mm sieve, and stored until characterization and application in the greenhouse experiment (Yin et al., 2024). The physicochemical properties of the two biochars are summarized in **Supplementary Table S3**. Representative Fourier-transform infrared (FTIR) spectra of the two biochars are shown in **Supplementary Figure S1**.

### 2.2 Experimental design and plant measurements

A 90-day greenhouse pot experiment was established using a factorial design comprising two biochar feedstocks (*A. palmeri* and *S. alterniflora*), three biochar application rates (1%, 3%, and 5%, w/w), and two nitrogen addition levels (0 and 30 kg N ha^−1^ yr^−1^). An unamended control without biochar or nitrogen was included separately. Thus, the experiment comprised 12 factorial biochar × rate × nitrogen treatments plus one unamended control, with eight independent replicates per treatment (**Supplementary Table S4**).

The nitrogen addition rate of 30 kg N ha^−1^ yr^−1^ was selected based on reported atmospheric nitrogen deposition in China and its potential increase under future deposition scenarios (Zhao et al., 2017). Nitrogen deposition was simulated using NH_4_NO_3_. The homogenized soil was stored in aluminum bags at 4°C during transportation to the greenhouse and was further homogenized one day after sampling. Approximately 10 kg of freshly collected wet soil was transferred into plastic trays (37 × 30 × 7.5 cm). The initial soil water content was approximately 39.8%. Biochar was thoroughly incorporated into the soil at rates of 1%, 3%, and 5% (w/w), calculated on a fresh-soil-weight basis.

The experiment was designed to examine the establishment and subsequent competitive performance of *F. bidentis* within plant communities emerging from a natural soil seed bank. *F. bidentis* was not artificially introduced or inoculated. The initial soil seed bank density was 3382 ± 453 seeds·m^−2^ (range: 1505–5009 seeds·m^−2^). Because the homogenized soil was thoroughly mixed before being distributed among the trays, the experimental units were established from the same pooled soil seed bank. Seeds were then allowed to germinate naturally under the respective biochar and nitrogen treatments. Importantly, the number of emerged seedlings was not artificially standardized after emergence, and all naturally emerging seedlings were retained until harvest. Thus, treatment effects on emergence and subsequent plant establishment were included in the observed plant-community responses. The species composition of the plant communities emerging in all treatments is provided in **Supplementary Table S5**. Importantly, seeds of *A. palmeri* and *S. alterniflora* were absent from the experimental soil and therefore did not constitute additional plant components of the experimental communities.

For nitrogen addition, NH_4_NO_3_ was dissolved in water and uniformly sprayed onto the soil surface to simulate atmospheric nitrogen deposition. The total nitrogen addition was calculated from the target annual deposition rate and the surface area of each tray. The annual amount of N corresponding to 30 kg N ha^−1^ yr^−1^ was 0.333 g N per tray and was divided equally between two applications on days 0 and 30. For each application, 0.1665 g N was applied to each tray. The detailed calculation is provided in **Supplementary Text S1**. Soil moisture was adjusted approximately every two days using deionized water to maintain approximately 70% of water-holding capacity immediately after irrigation. This moisture level was selected based on preliminary experiments showing that it provided suitable conditions for seed germination and plant establishment while avoiding excessive moisture. Because biochar amendments can alter soil water retention, the 70% water-holding capacity target represents the soil moisture immediately following irrigation rather than a constant moisture level maintained throughout the two-day interval. Greenhouse conditions were maintained at 22–25°C under a 12-h photoperiod (5000 lux). Trays were randomly arranged and repositioned twice during the experiment to minimize positional effects.

At harvest, plant height and stem diameter were measured using a ruler and digital vernier caliper, respectively. The abundance of each species was determined by counting all individuals within each tray. Aboveground biomass was harvested, rinsed with deionized water, oven-dried at 65°C to constant weight, and weighed. Because emergence was allowed to proceed naturally and was not standardized before harvest, differences in final abundance and biomass may reflect both treatment effects on emergence/establishment and subsequent plant performance. Accordingly, the resulting invasion indices were interpreted as integrated responses of plant establishment and performance rather than as measurements of competitive interactions independent of emergence.

### 2.3 Invasion-related indices

To quantify treatment effects on community invasibility, invasion resistance, and the competitive performance of *F. bidentis*, four invasion-related indices were calculated, including the community invasibility index (CII, Equation 1), invasion resistance index (IRI, Equation 2), competitive advantage index (CAI, Equation 3), and relative invasiveness index (RII, Equation 4). CII and IRI were used to characterize the susceptibility and resistance of the plant community to invasion, respectively, whereas CAI and RII were used to characterize the relative competitive performance of *F. bidentis* (Wang et al., 2020, 2021). The calculation formulas are as follows:

(1) CII=1−(MaxPi−Pi)

(2) $$IRI=1-\frac{\sum PiXi}{\sum PnXn}$$

(3) $$CAI=\frac{\sum PiXi}{\sum PnXn}$$

where *Pi* is the relative abundance of *F. bidentis* in each tray, and *MaxPi* is the maximum relative abundance observed across all invaded trays. *Pn* represents the relative abundance of native species. *Xi* and *Xn* denote the arithmetic mean of aboveground biomass, abundance, plant height, and stem diameter of *F. bidentis* and native species, respectively. Notably, IRI and CAI are opposite in sign, providing complementary perspectives on the intensity of plant invasion.

Relative invasiveness (RII) was calculated as:

(4) $$RII=\frac{Bi}{MaxBi}$$

where *Bi* is the total aboveground biomass of *F. bidentis* in each tray, and *MaxBi* is the maximum biomass recorded among all invaded trays (Wang et al., 2020, 2021).

### 2.4 Plant community analysis

Species richness, Shannon–Wiener diversity, and Simpson diversity indices were calculated from species abundance data using the vegan package, whereas Faith’s phylogenetic diversity (PD) was calculated using the picante package. Differences in community composition among treatments were assessed using permutational multivariate analysis of variance (PERMANOVA) implemented with the *adonis* function in the vegan package. To characterize the performance-based niche differentiation between native and invasive plants under biochar and nitrogen exposure, we estimated niche breadth and niche overlap using Levins’ niche theory framework. Aboveground growth indicators were used as the response variables defining species performance across these treatment states. Niche breadth and niche overlap were calculated using the *niche.width* and *niche.overlap* functions implemented in the spaa package (Zhang, 2016). Before niche-overlap calculation, species abundance data were standardized to relative abundance within each treatment matrix, thereby ensuring that the overlap estimates reflected the proportional distribution of species performance across the experimental conditions.

A phylogenetic tree for all plant species recorded across the experiment was constructed using the V.PhyloMaker2 package (Jin and Qian, 2019). In total, 18 plant species were recorded across the experimental communities, and this complete observed species pool was used for phylogenetic community analyses. To characterize phylogenetic turnover among plant communities, we calculated beta mean pairwise distance (*β*MPD) and beta mean nearest taxon distance (*β*MNTD). Their standardized effect sizes were expressed as the beta nearest taxon index (*β*NTI) and beta net relatedness index (*β*NRI), respectively. To infer the relative importance of deterministic versus stochastic processes in community assembly. Values of |*β*NTI| or |*β*NRI| > 2 were interpreted as indicating stronger deterministic assembly processes, whereas values within ±2 were interpreted as being more consistent with stochastic processes. The phylogenetic metrics were calculated using the *ses.mpd* and *ses.mntd* functions in the *picante* package (Kembel et al., 2010) and the *bmntd*, *bmpd*, *bNTIn.p*, and *bNRIn.p* functions in the *iCAMP* package (Ning et al., 2020) within R.

### 2.5 Statistical analyses

The effects of biochar feedstock, biochar application rate, nitrogen addition, and their interactions on plant growth traits, invasion-related indices, and community diversity were evaluated using three-way analysis of variance (ANOVA). Before ANOVA, model residuals were examined for normality using the Shapiro–Wilk test and normal probability plots, and homogeneity of variance was assessed using Levene’s test. When necessary, response variables were log- or square-root transformed to improve conformity with model assumptions. The transformed or untransformed data were then used for subsequent analyses. When a significant main effect was detected, estimated marginal means were compared using appropriate *post hoc* contrasts. Comparisons of each biochar–nitrogen treatment with the unamended control were performed using Dunnett-adjusted contrasts, whereas comparisons between biochar-only and corresponding biochar + nitrogen treatments were conducted using planned pairwise contrasts within the same biochar feedstock and application rate. For significant interactions, simple-effect analyses were conducted to evaluate the effect of one factor at each level of the interacting factors, rather than interpreting the main effects independently. Multiplicity was controlled using the appropriate adjustment for each family of pairwise contrasts, and adjusted *p*-values < 0.05 were considered statistically significant. All statistical analyses were performed in R version 4.6.1 (R Core Team, Vienna, Austria), and figures were generated using the ggplot2 package. All reported values are presented as mean ± standard error.

## 3 Results

### 3.1 Biochar dose and nitrogen differentially regulate invasive and native plant performance

Biochar feedstock, biochar application rate, and nitrogen addition significantly influenced the growth performance of both *F. bidentis* and the native plant community, but their effects depended on the response variable and the interactions among factors (**Figure 1**; **Supplementary Table S6**). For *F. bidentis*, biochar feedstock and biochar application rate significantly affected aboveground biomass and abundance, whereas the main effects of biochar application rate and nitrogen addition were not significant for plant height or stem diameter. However, significant interactions between biochar application rate and nitrogen addition were detected for both plant height and stem diameter.

**Figure 1:**
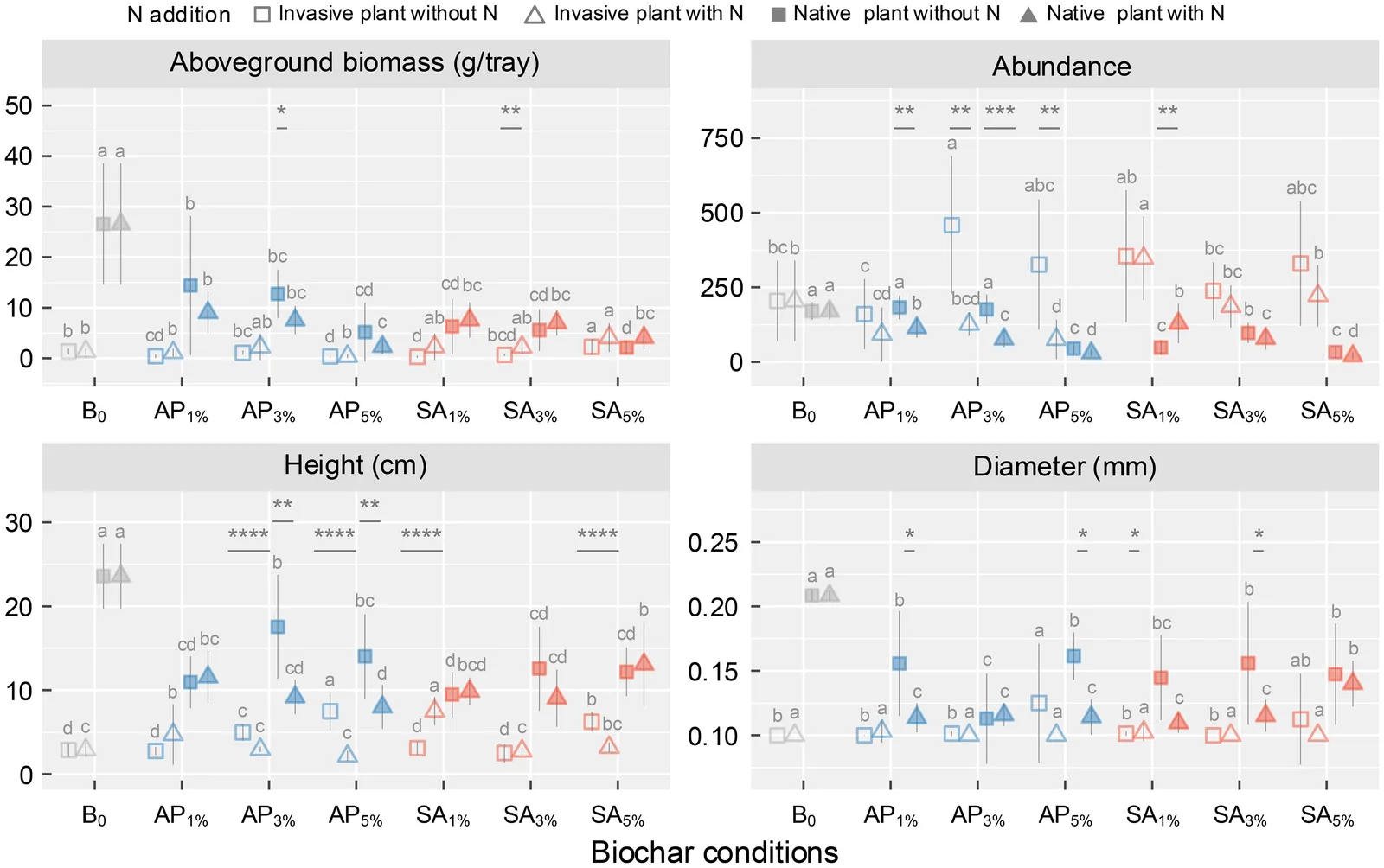
Regulation of biochar feedstock, biochar application rate, and nitrogen (N) addition on aboveground biomass, abundance, height, and stem diameter of invasive and native plants. Treatments: B0, unamended control (without biochar or nitrogen); AP_1%_, AP_3%_, and AP_5%_, *Amaranthus palmeri*-derived biochar applied at 1%, 3%, and 5% (w/w), respectively; SA_1%_, SA_3%_, and SA_5%_, *Spartina alterniflora*-derived biochar applied at 1%, 3%, and 5%, respectively. Values are means ± SE. Different lowercase letters indicate significant differences among biochar treatments within the same nitrogen level (LSD test), whereas asterisks indicate significant effects of nitrogen addition within the same biochar treatment (*p < 0.05; **p < 0.01; ***p < 0.001). Unless otherwise stated, the abbreviations, treatment codes, and statistical annotations defined here apply to all subsequent images.

Under biochar-only conditions, the response of *F. bidentis* varied with biochar feedstock and application rate. Compare with the no-biochar, no-nitrogen control (B_0_, percentage change (%) = [(mean of treatment − mean of B_0_)/mean of B_0_] × 100%, the same applies below), 1% AP-biochar reduced the aboveground biomass of *F. bidentis* by 68.5% (*p* < 0.05, LSD test), whereas, 3% AP-biochar increased invasive plant abundance and height by 123.9% and 71.8%, respectively (*p* < 0.05, LSD test). At 5% AP-biochar, aboveground biomass was reduced by 72.4%, whereas plant height and stem diameter increased by 159.1% and 25.0%, respectively (*p* < 0.05, LSD test). Similarly, 1% SA-biochar reduced invasive aboveground biomass by 78.9%, whereas 5% SA-biochar increased invasive aboveground biomass and height by 67.3% and 115.5%, respectively (*p* < 0.05, LSD test).

In contrast, native plants exhibited a consistently negative response to biochar addition regardless of feedstock. Under AP-biochar, native aboveground biomass, plant height, and stem diameter declined by 45.9%–80.6%, 25.7%–53.6%, and 22.6%–45.9%, respectively (*p* < 0.05, LSD test). Likewise, SA-biochar significantly reduced all measured native plant traits, with aboveground biomass declining by 76.4%–92.1%, abundance by 43.0%–80.6%, plant height by 46.7%–59.9%, and stem diameter by 25.3%–30.6% (all *p* < 0.05, LSD test). Collectively, these results indicate that biochar alone primarily weakened native plant performance, while its effects on *F. bidentis* shifted from inhibition at low biochar application rates to partial stimulation at medium and high rates.

Nitrogen addition substantially modified these biochar responses, although the magnitude and direction of the effects depended on biochar feedstock and application rate. Relative to the corresponding biochar-only treatments (Percentage change (%) = [(mean of biochar + N − mean of corresponding biochar-only treatment)/mean of corresponding biochar-only treatment] × 100%, the same applies below), nitrogen co-addition with AP-biochar generally suppressed invasive plant performance while further reducing several native plant traits. Under the 3% AP-biochar condition, nitrogen addition decreased invasive abundance and plant height by 72.4% and 40.9%, respectively (*p* < 0.05, t-test), accompanied by reductions in native aboveground biomass (40.7%), abundance (56.5%), and height (47.6%, *p* < 0.05, t-test). Nitrogen addition also reduced invasive plant height by 71.4% under 5% AP-biochar and decreased native plant height and stem diameter by 43.0% and 29.2%, respectively (*p* < 0.05, t-test).

Responses under SA-biochar were more variable. Nitrogen co-addition increased invasive plant height by 142.8% under 1% SA-biochar and increased native plant abundance by 166.1%, although native stem diameter decreased by 24.2% (*p* < 0.05, t-test). Under 3% SA-biochar, invasive aboveground biomass increased by 235.3%, whereas native stem diameter declined by 26.1% (*p* < 0.05, t-test). In contrast, invasive plant height decreased by 49.0% under 5% SA-biochar with nitrogen addition (*p* < 0.05, t-test). Overall, nitrogen altered the biochar response in a feedstock- and dose-dependent manner, with AP-biochar tending to suppress invasive plant growth under nitrogen enrichment, whereas responses to SA-biochar were less consistent.

### 3.2 Nitrogen offsets biochar-induced increases in invasion potential and restores community resistance

Biochar application significantly altered invasion dynamics by modifying community invasibility and invasion resistance, as well as competitive advantage, and the relative invasiveness of *F. bidentis* (**Figure 2**; **Supplementary Table S6**). Under biochar-only conditions, both AP- and SA-derived biochars generally increased community susceptibility to invasion while reducing invasion resistance. For AP-biochar, 1% application reduced the relative invasiveness index by 0.21, whereas 3% AP-biochar increased the community invasibility index by 0.20 (*p* < 0.05, LSD test). The strongest response occurred under 5% AP-biochar, where the community invasibility index increased by 0.34, the competitive advantage index increased by 72, and the relative invasiveness index decreased by 0.20 (*p* < 0.05, LSD test).

**Figure 2:**
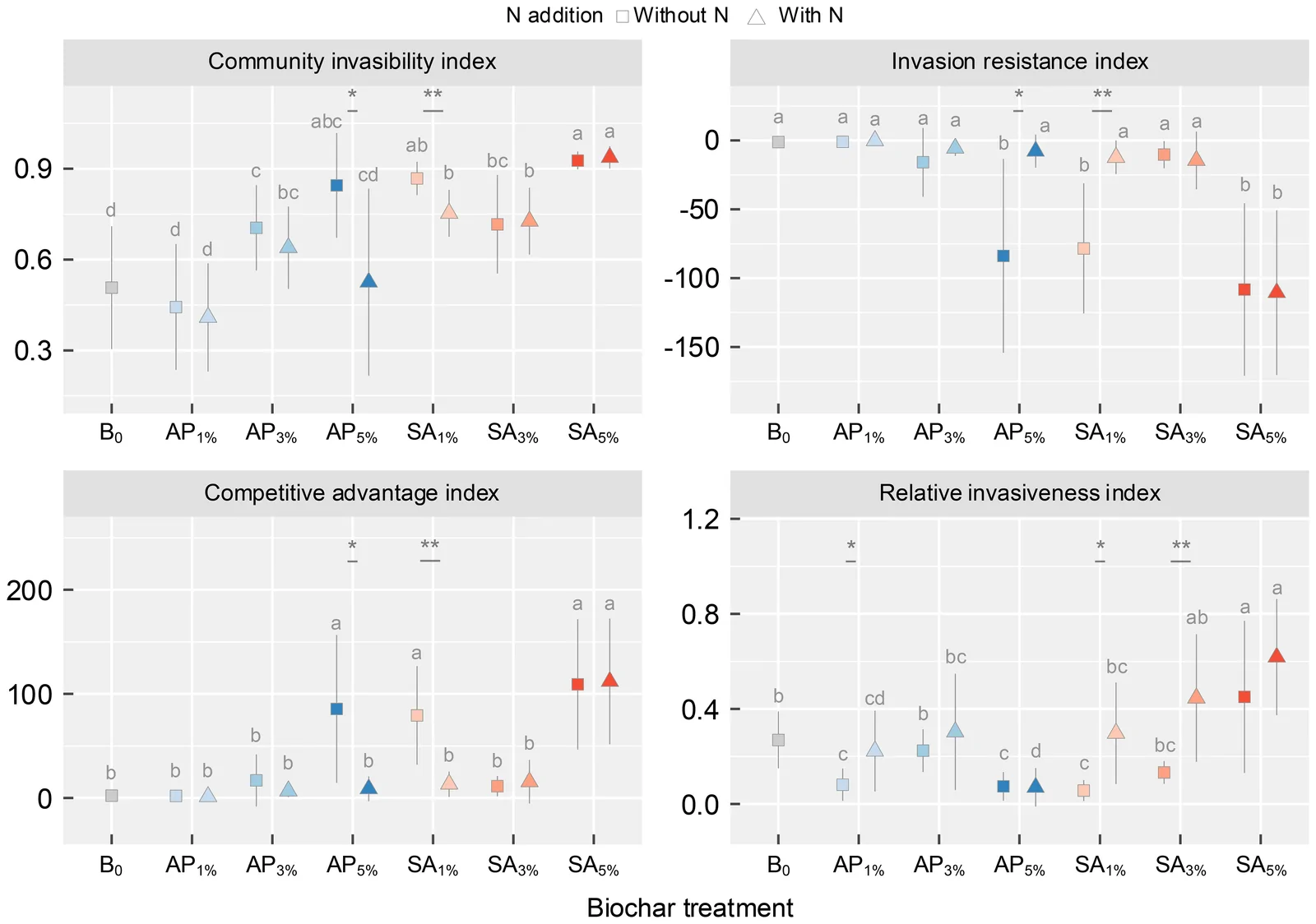
Reshape of heterospecific invasive plant-derived biochar and nitrogen addition on community invasibility, invasion resistance, competitive advantage, and relative invasiveness of *Flaveria bidentis*.

A similar pattern was observed under SA-biochar. Compared with the no-biochar, no-nitrogen condition, 1% SA-biochar increased the community relative invasibility index by 0.36 and the competitive advantage index by 77 but reduced the relative invasiveness index by 0.21 (*p* < 0.05, LSD test). Community invasibility also increased under 3% SA-biochar, whereas 5% SA-biochar produced the greatest invasion response, increasing community relative invasibility index by 0.42, competitive advantage index by 107, and relative invasiveness index by 0.18 (*p* < 0.05, LSD test). These results indicate that biochar-induced increases in invasion were primarily associated with reduced resistance of the native community rather than sustained enhancement of the intrinsic competitive ability of *F. bidentis*.

Nitrogen addition substantially modified these invasion responses. Compared with the corresponding biochar-only treatments, nitrogen co-addition with AP-biochar consistently enhanced community resistance and weakened invasion potential. Under 1% AP-biochar, the relative invasiveness index decreased by 0.16 (*p* < 0.05, t-test). More pronounced effects were observed under 5% AP-biochar, where nitrogen reduced the community relative invasibility index by 0.32 and the competitive advantage index by 65 (*p* < 0.05, t-test). These findings demonstrate that nitrogen addition effectively offset the increase in invasion potential induced by AP-derived biochar by suppressing the competitive advantage of *F. bidentis* and strengthening community resistance.

Nitrogen exerted comparable, although weaker, effects under SA-derived biochar. Relative to biochar alone, nitrogen reduced the competitive advantage index by 66 under 1% SA-biochar, while 5% SA-biochar increased the relative invasiveness index by 0.31 (*p* < 0.05, t-test). Collectively, these findings suggest that nitrogen enrichment partially mitigated the invasion-promoting effects of invasive plant-derived biochar, particularly when AP-derived biochar was applied.

### 3.3 Biochar–nitrogen interactions reshape plant community diversity and composition

Biochar application significantly influenced plant community diversity, although its effects depended on biochar feedstock, biochar application rate, and nitrogen addition (**Figure 3a**; **Supplementary Table S7**). Compared with the no-biochar, no-nitrogen condition, biochar alone generally reduced species diversity across most treatments (*p* < 0.05, LSD test), whereas low biochar application rates (1% AP- and 1% SA-biochar) produced little or no significant change (*p* > 0.05, LSD test). These results indicate that biochar application alone tended to simplify community structure, particularly at medium and high biochar application rates.

**Figure 3:**
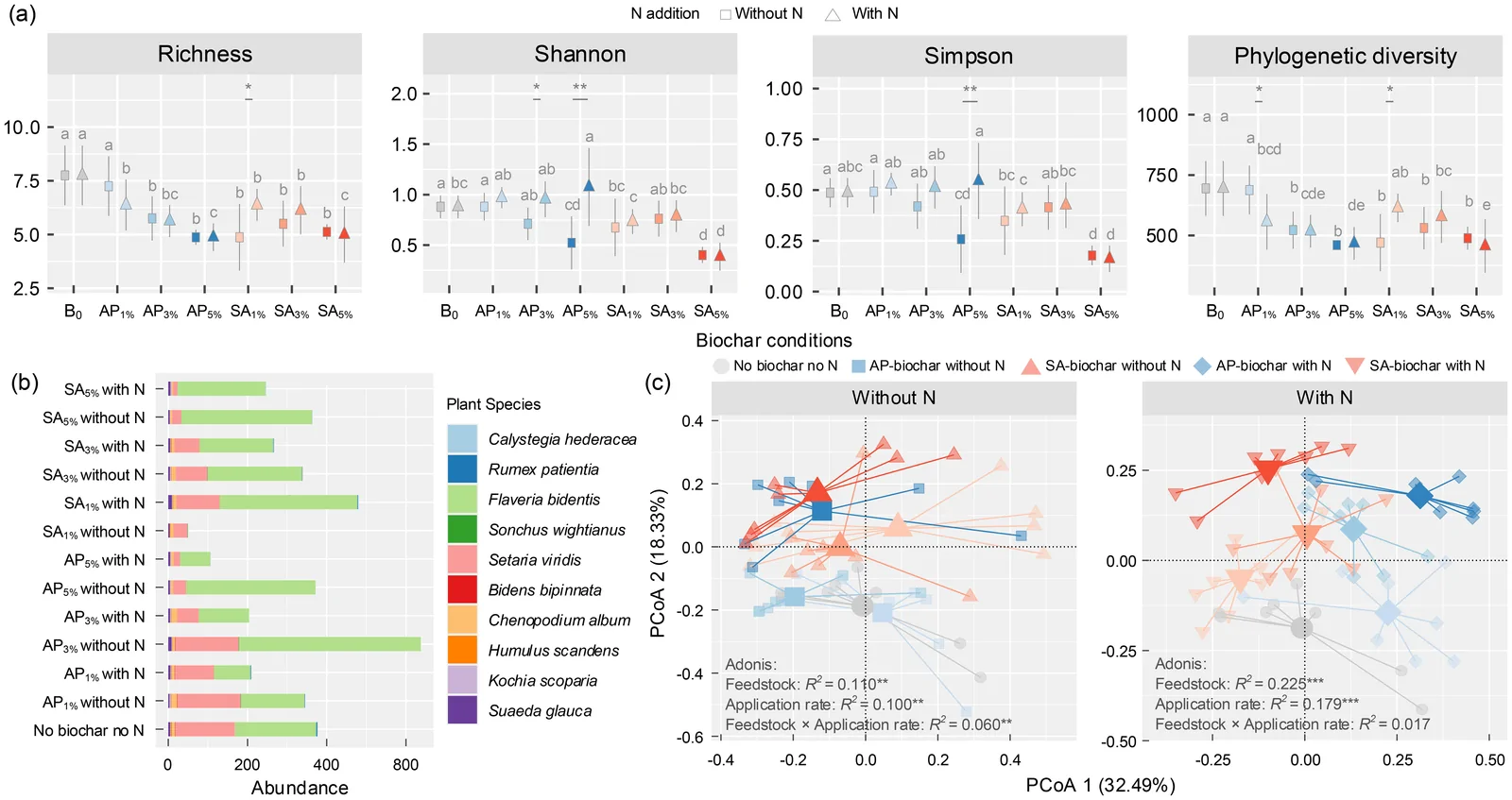
Alteration of biochar feedstock, biochar application rate, and nitrogen addition on **(a)** plant community α-diversity, **(b)** abundance of dominant plant species, and **(c)** Bray–Curtis dissimilarity of plant communities.

Nitrogen addition substantially modified these responses. Compared with the corresponding biochar-only treatments, nitrogen co-addition with AP-derived biochar generally enhanced community diversity despite a modest reduction in phylogenetic diversity under 1% AP-biochar (19.15%, *p* < 0.05, t-test). Under 3% AP-biochar, Shannon diversity increased by 34.1%, whereas 5% AP-biochar produced the greatest improvement, increasing Shannon and Simpson diversity by 106.2% and 110.8%, respectively (*p* < 0.05, t-test; **Figure 3a**). Similarly, nitrogen addition combined with 1% SA-biochar increased species richness by 30.8% and Faith’s phylogenetic diversity by 30.6% (*p* < 0.05, t test). Overall, nitrogen enrichment largely offset the diversity loss induced by biochar alone, with the strongest positive effects observed under AP-derived biochar at the highest biochar application rate.

Similar to the pronounced effects on community diversity, biochar or nitrogen addition markedly influenced the plant community composition (**Figure 3b**; **Supplementary Table S8**). Under biochar alone conditions, biochar feedstock, biochar application rate, and their interaction all significantly affected community composition (Adonis: *R^2^* = 0.06-0.11, all *p* < 0.01; **Figure 3c**). With N addition, the effects of biochar feedstock (*R^2^* = 0.225, *p* < 0.001) and biochar application rate were further strengthened (*R^2^* = 0.179, *p* < 0.001), whereas their interaction was not significant. Overall, different biochar treatments, particularly AP-biochar and SA-biochar, showed clear community differentiation.

### 3.4 Biochar and nitrogen modify ecological niche differentiation and phylogenetic community assembly

Biochar feedstock, biochar application rate, and nitrogen enrichment jointly altered ecological niche characteristics of *F. bidentis* and the dominant native plant species (**Figure 4**). Across biochar-only treatments, the niche breadth of *F. bidentis* generally declined, decreasing by 0.37 under 1% AP-biochar, 0.06 under 5% AP-biochar, and 2.07 under 1% SA-biochar (**Figure 4a**). Nitrogen addition further constrained the ecological niche of the invasive species, reducing niche breadth by 1.11 and 1.53 under 1% and 5% AP-biochar, respectively. These results indicate that nitrogen enrichment strengthened the restrictive effects of biochar on the ecological space occupied by *F. bidentis*.

**Figure 4:**
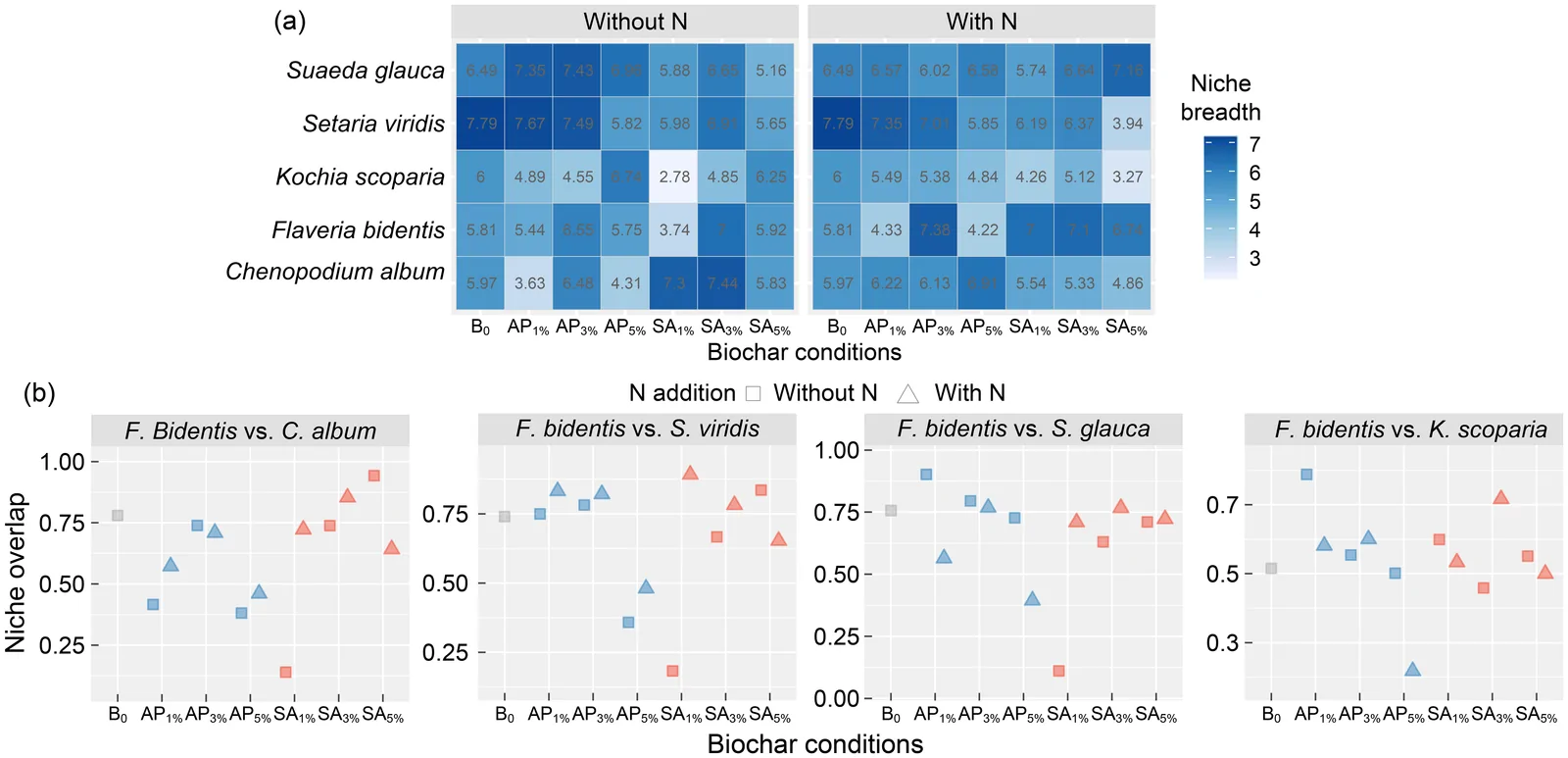
Modification of biochar feedstock, biochar application rate, and nitrogen addition on **(a)** niche breadth of the five dominant plant species and **(b)** niche overlap between *Flaveria bidentis* and dominant native species.

Patterns of niche overlap further demonstrated that biochar altered competitive relationships between the invasive and native species (**Figure 4b**). *F. bidentis* exhibited relatively high niche overlap with the dominant native species *S. viridis* (0.18–0.84), whereas overlap with *C. album* remained comparatively low (0.14–0.94). Compared with the no-biochar, no-nitrogen condition, AP-derived biochar substantially reduced niche overlap between *F. bidentis* and *C. album*, declining by 0.36 under 1% AP-biochar and 0.40 under 5% AP-biochar. Relative to the corresponding biochar-only treatments, nitrogen co-addition reduced performance-based niche overlap between *F. bidentis* and the dominant native *S. glauca* by 0.12–0.34, but increased overlap between *F. bidentis* and the dominant native *S. viridis* by 0.04–0.12.

Biochar and nitrogen addition altered several measures of phylogenetic *β*-diversity. Under biochar-only conditions, increasing AP- and SA-biochar application rates generally decreased βMPD (**Figure 5**). A similar declining trend was observed for βNTI under AP-biochar, with βNTI decreasing by 0.87 and 0.83 at 3% and 5% AP-biochar, respectively (*p* < 0.05, LSD test). However, these βNTI values remained within the ±2 threshold across all treatments, indicating no strong deterministic signal in phylogenetic community assembly.

**Figure 5:**
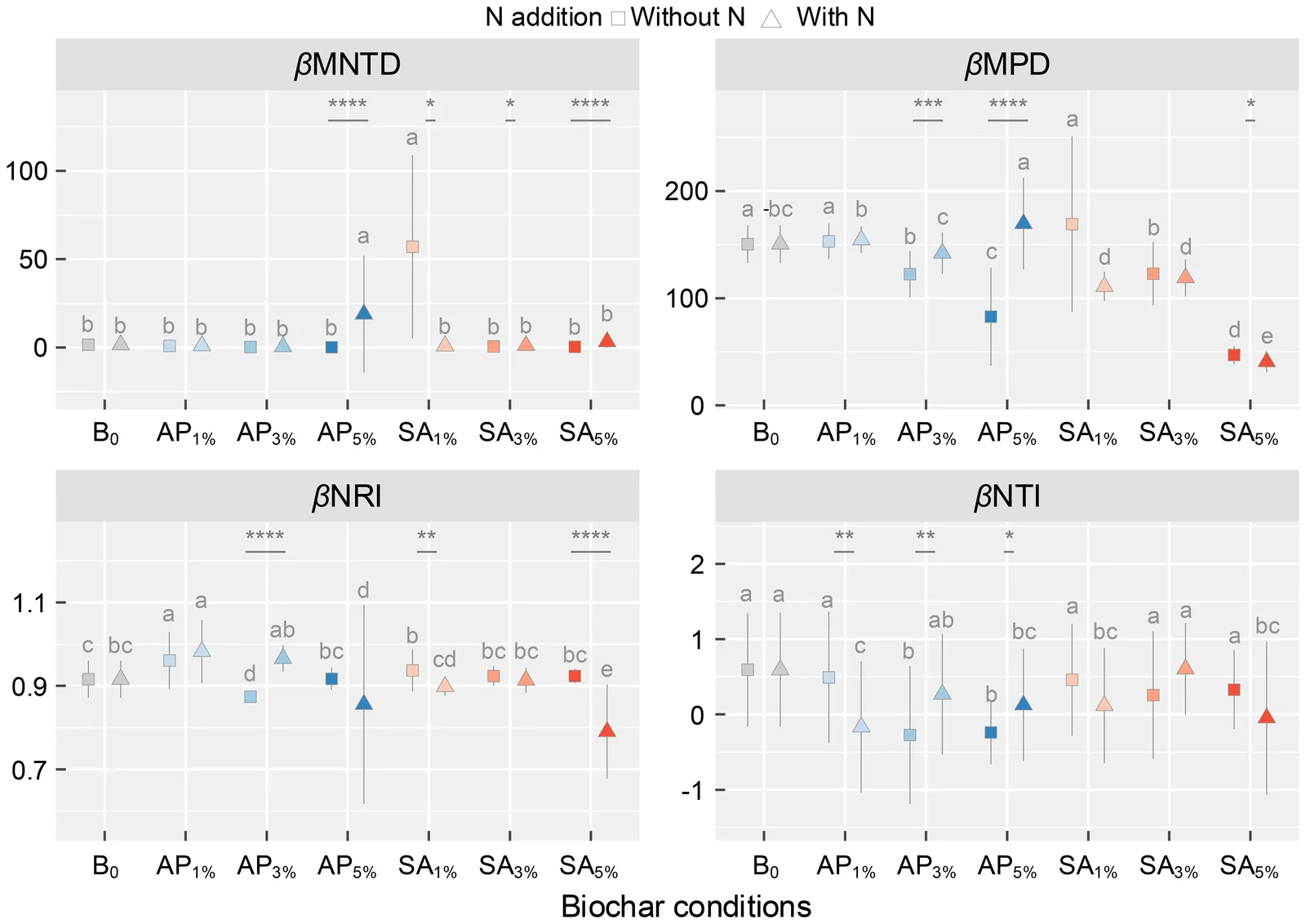
Interaction effects of biochar feedstock, biochar application rate, and nitrogen addition on plant community phylogenetic *β*-diversity.

Nitrogen addition moderated the effects of AP-biochar on phylogenetic β-diversity. Relative to the corresponding biochar-only treatments, nitrogen increased βMPD by 19.56 and 86.78 under 3% and 5% AP-biochar, respectively, and increased βNTI by 0.37 under 5% AP-biochar (*p* < 0.05, t-test). Thus, nitrogen addition partially alleviated the declining trends in βMPD and βNTI observed with increasing AP-biochar application. In contrast, the responses to SA-biochar were less consistent, with nitrogen addition producing variable changes in βMPD and βNTI across application rates. In general, nitrogen addition primarily modified the phylogenetic turnover associated with AP-biochar, whereas its effects under SA-biochar were comparatively weak and variable.

Overall, these findings reveal that biochar application, particularly when combined with nitrogen enrichment, constrained the ecological niche of *F. bidentis*, reduced niche overlap between invasive and native species, and reshaped phylogenetic community assembly. Together with the observed changes in community diversity and composition, these results suggest that biochar–nitrogen interactions could influence invasion outcomes by modifying both ecological niche relationships and phylogenetic structure.

## 4 Discussion

### 4.1 Invasive plant-derived biochar alters invasion trajectories by reshaping competitive interactions and weakening native community resistance

Our first hypothesis predicted that heterospecific invasive plant-derived biochar would facilitate *F. bidentis* invasion by enhancing invader performance while suppressing native plant communities. This hypothesis was partially supported. Rather than exerting a uniformly facilitative or inhibitory effect, invasive plant-derived biochar generated highly context-dependent outcomes determined by biochar feedstock identity and application rate. In particular, medium- to high-dose AP-biochar and SA-biochar shifted competitive interactions toward *F. bidentis*, whereas low-dose biochar imposed broad growth constraints on both invasive and native plants. These findings indicate that the ecological consequences of invasive plant-derived biochar cannot be inferred solely from its feedstock origin. Instead, invasion outcomes depend on how individual biochar amendments modify the competitive balance between the invader and resident community.

Low biochar application rates suppressed the aboveground biomass of *F. bidentis*, but simultaneously reduced several growth traits of native plants, indicating a largely nonselective stress response rather than targeted inhibition of the invader. For example, 1% AP-biochar and 1% SA-biochar decreased *F. bidentis* biomass by 68.5% and 78.9%, respectively, while also substantially reducing native plant biomass, height, and stem diameter (**Figure 1**). Such short-term suppression is consistent with physicochemical disturbances associated with freshly produced biochar, particularly in saline–alkaline soils where plants already experience substantial osmotic and ionic constraints. Alkaline ash and soluble salts released from biochar may modify soil pH, electrical conductivity, osmotic conditions, and nutrient availability (Hussain et al., 2022; Mikajlo et al., 2024; Zhang et al., 2026a). In addition, biochar surfaces may temporarily adsorb mineral nutrients, whereas residual phenolic compounds, polycyclic aromatic hydrocarbons, and other pyrolysis-derived molecules may constrain seedling establishment and early plant growth (Thomas et al., 2024; Zhang et al., 2025a; Wang et al., 2026a). The physicochemical characterization of the two biochars further confirms that AP- and SA-biochar should not be considered functionally equivalent amendments. Their differences in pH, C/N ratio, and elemental composition provide a material basis for feedstock-dependent ecological responses (**Supplementary Table S2**). These properties can influence nutrient sorption, ion exchange, water retention, and the release of soluble constituents. Nevertheless, because soluble salt release, residual phenolics and polycyclic aromatic hydrocarbons, cation-exchange capacity, pore-scale properties, and post-treatment soil pH, electrical conductivity, and nutrient availability were not quantified, the respective contributions of these mechanisms cannot be disentangled in the present experiment. Accordingly, the observed feedstock effect should be interpreted as evidence of a response associated with differences in biochar properties rather than direct evidence for any specific physicochemical driver.

In contrast to the generalized suppression observed at low application rates (1%), 3% AP-biochar and 5% SA-biochar promoted one or more growth attributes of *F. bidentis* while strongly suppressing native plant performance (**Figure 1**). This shift suggests that some biochar treatments may alter competitive asymmetry rather than uniformly improve or impair plant growth. Feedstock-specific differences in mineral composition and elemental stoichiometry may contribute to contrasting plant responses to biochar (Bieser et al., 2022; Fartyal et al., 2025; Li et al., 2025c). Biochar-mediated changes in root-zone conditions may also influence plant–soil interactions, including nutrient acquisition and microbial associations (Dwibedi et al., 2022; Shen et al., 2024; Liu et al., 2026b). Previous studies have reported relatively high arbuscular mycorrhizal fungal colonization in *F. bidentis* compared with the native species *S. viridis* (Zhang et al., 2017; Pu et al., 2026). Thus, altered soil conditions could potentially produce unequal benefits among species. Likewise, the documented allelopathic potential of *F. bidentis* may further intensify competitive asymmetry once environmental conditions permit rapid invader growth (Kamel et al., 2025; Mohammed et al., 2025). However, neither arbuscular mycorrhizal fungal colonization nor microbial communities, nutrient cycling, plant nutrient uptake, or allelochemical concentrations were measured in this study. These processes should therefore be regarded as plausible, complementary explanations rather than experimentally demonstrated mechanisms.

Importantly, the invasion-promoting effects of biochar were expressed more clearly at the community level than through a consistent increase in the absolute performance of *F. bidentis*. Biochar-only treatments generally decreased community diversity and reduced invasion resistance, as indicated by increased community relative invasibility and/or competitive advantage, and decreases in invasion resistance (**Figures 2**, **3**). These results suggest that certain biochar treatments may increase invasion vulnerability primarily by impairing native plant performance and weakening biotic resistance. Reduced native biomass and stature can alter access to light, space, and soil nutrient resources, thereby changing the competitive environment experienced by the invader (Feng et al., 2021; Li et al., 2025c). Thus, the invasion consequences of biochar cannot be evaluated solely from changes in *F. bidentis* biomass. Native community responses are equally important because invasion risk is inherently determined by interactions between invader performance and resident community resistance.

Biochar also modified invasion processes by restructuring ecological niche relationships. Biochar alone frequently reduced the niche breadth of *F. bidentis*, with the largest decline observed under 1% SA-biochar (−2.07; **Figure 4**). A narrower niche breadth does not, however, necessarily imply lower invasion risk. Rather, it indicates that the species occupied a more restricted position along the resource or habitat gradients represented by the community. Biochar may have redistributed resources across microsites and modified interspecific interactions, allowing some species to expand their realized niches while constraining others (Bitarafan et al., 2019; Li et al., 2025d; Sun et al., 2026). The distinct physicochemical properties of SA-biochar may have contributed to this response, although the specific mechanisms remain unresolved because post-treatment soil properties were not measured.

Changes in performance-based niche overlap further indicated that biochar altered the similarity of plant performance responses across the experimental conditions. For example, AP-biochar reduced the performance-based niche overlap between *F. bidentis* and *Chenopodium album* by up to 0.40, indicating increasingly divergent responses to the modified resource environment (Yin et al., 2024; Ghorbani et al., 2026). Because *F. bidentis* is a C4 species with efficient resource acquisition under warm and relatively dry conditions, whereas *C. album* is a C3 species that may respond differently to nutrient availability, changes in soil moisture, nitrogen availability, and pH could shift the two species toward different portions of the available resource gradient (Tsujimoto and Hikosaka, 2021; Oono et al., 2022; Baros et al., 2024; Bernardo et al., 2026). Such differentiation may reduce direct niche overlap while still permitting *F. bidentis* to dominate favorable portions of the environmental gradient.

Changes in performance-based niche structure were not consistently accompanied by parallel shifts in phylogenetic β-diversity (**Figure 5**), indicating a partial decoupling between invader performance responses and phylogenetic community turnover. Biochar treatments that altered *F. bidentis* performance-based niche breadth did not necessarily generate equivalent changes in βMPD, βMNTD, βNTI, or βNRI. Moreover, most βNRI and βNTI values remained within the ±2 threshold, providing limited evidence for strong deterministic assembly processes. Thus, the observed phylogenetic patterns are better interpreted as changes in phylogenetic turnover rather than clear evidence of intensified environmental filtering. Together, these results indicate that biochar effects on plant performance and phylogenetic community structure may operate through partly independent pathways. Accordingly, our first hypothesis was only partially supported: invasive plant-derived biochar did not universally promote *F. bidentis*, but specific feedstock–dose combinations altered plant performance and community-level invasion responses.

In this study, niche breadth and niche overlap were used to characterize the performance-based niche of native and invasive plants across the experimentally imposed biochar and nitrogen treatments. Rather than quantifying complete resource-use niches, these indices captured differences in plant growth responses and response similarity under the experimental conditions. We acknowledge that this represents a one-dimensional niche characterization because environmental dimensions such as soil nutrients, moisture, and salinity were not incorporated into the niche matrix. Therefore, the observed niche breadth and overlap should not be interpreted as comprehensive measures of resource partitioning or interspecific competition. Nevertheless, this approach provides a direct assessment of how biochar and nitrogen treatments modify the relative performance space of *F. bidentis* and native plants. Future studies integrating plant performance with multidimensional environmental measurements will provide a more comprehensive evaluation of resource use and niche differentiation.

### 4.2 Nitrogen addition reshapes biochar-mediated invasion outcomes in a feedstock- and dose-dependent manner

Our second hypothesis predicted that nitrogen addition would alleviate the detrimental effects of heterospecific invasive plant-derived biochar on plant community structure. The results partially supported this hypothesis. Nitrogen addition did not uniformly mitigate biochar-mediated invasion. Instead, it redirected plant-community responses in a strongly feedstock- and dose-dependent manner. Nitrogen co-addition generally counteracted the invasion-promoting effects of AP-biochar, whereas low- and moderate- dose SA-biochar combined with nitrogen frequently enhanced the absolute growth of *F. bidentis*. Thus, nitrogen enrichment did not simply strengthen or weaken biochar effects; rather, it modified the resource environment within which invasive and native plants competed.

The contrasting responses of *F. bidentis* to AP- and SA-biochar under nitrogen enrichment may reflect differences in their interactions with soil nutrient dynamics and rhizosphere processes (Antonangelo et al., 2025; Wan et al., 2025). Unless otherwise stated, comparisons below refer to biochar–nitrogen co-addition relative to the corresponding biochar-only treatment. AP-biochar likely retained a proportion of added nitrogen through adsorption while simultaneously modifying soil pH, salinity, and nutrient stoichiometry, thereby reducing nitrogen accessibility to *F. bidentis* (Haris et al., 2024; Liao et al., 2024; Wan et al., 2025). Nitrogen enrichment may also shift plant nutrient limitation from nitrogen to phosphorus or other essential elements, constraining the capacity of *F. bidentis* to convert additional nitrogen into biomass (Zhang et al., 2025c; Mmbaga and Materu, 2026). Consistent with this interpretation, nitrogen addition further reduced the abundance, height, and aboveground biomass of *F. bidentis* under the 3% and 5% AP-biochar treatments.

A complementary mechanism is that nitrogen addition may intensify microbial competition for limiting nutrients, particularly under biochar-amended saline–alkaline conditions. Nitrogen enrichment can stimulate microbial growth and nutrient immobilization, potentially increasing microbial demand for phosphorus and micronutrients (Gao et al., 2026; Lv et al., 2026). Biochar may further modify microbial habitat and resource accessibility through its porous structure, surface chemistry, and mineral constituents (Liu et al., 2026a). Under such conditions, microbial immobilization could temporarily restrict plant access to nutrients and create a “microbial resource trap”. This mechanism could suppress both native plants and *F. bidentis*, but its consequences may be particularly pronounced for a fast-growing invader with high absolute nutrient requirements. Differences in biochar porosity, surface functional groups, and mineral composition between AP- and SA-biochar could further regulate microbial colonization and nutrient competition, potentially contributing to their contrasting effects (Thomas et al., 2024; Wang et al., 2026a). However, because microbial biomass, microbial nutrient immobilization, and soil phosphorus and micronutrient dynamics were not quantified in this study, this mechanism remains a plausible complementary explanation rather than a demonstrated causal pathway. Future experiments integrating microbial biomass, extracellular enzyme activity, nutrient fluxes, and plant nutrient uptake will be necessary to test this hypothesis directly. In contrast, the response of *F. bidentis* to SA-biochar under nitrogen enrichment was nonlinear. Nitrogen co-addition markedly increased plant height under 1% SA-biochar and aboveground biomass under 3% SA-biochar, whereas plant height declined under 5% SA-biochar (**Figure 1**). These responses suggest that low-to-moderate SA-biochar application may have created a rhizosphere environment favorable to invasive plant growth, whereas higher biochar loading imposed physiological constraints through increased salinity, altered ion balance, or excessive alkalinity (Jin et al., 2026; Shen et al., 2026). Arbuscular mycorrhizal fungi may further contribute to these contrasting responses. Moderate SA-biochar application may improve soil aeration, moisture retention, and nutrient availability, thereby enhancing arbuscular mycorrhizal fungi -mediated nutrient acquisition and promoting the competitive performance of *F. bidentis* (Li et al., 2025b; Pu et al., 2026; Wang et al., 2026b). Conversely, AP-biochar may generate rhizosphere conditions less favorable for arbuscular mycorrhizal fungi colonization or functioning (Nirukshan et al., 2022; Wang et al., 2026b). Because arbuscular mycorrhizal fungi colonization and community composition were not measured in the present study, however, this interpretation remains tentative. Nitrogen addition also substantially altered invasion resistance at the community level. Among the tested treatments, 5% AP-biochar combined with nitrogen most effectively reduced community invasibility and increased invasion resistance (**Figure 2**), indicating a shift in competitive balance toward the native community. This response may result from stronger modification of nitrogen availability and rhizosphere nutrient dynamics at the higher AP-biochar rate, which constrained resource acquisition by *F. bidentis* and reduced its competitive asymmetry with native species (Bai et al., 2026; Zhang et al., 2026a). The microbial resource-trap mechanism proposed above may provide an additional explanation if nitrogen-induced microbial immobilization was stronger under AP-biochar. Such microbial competition could further restrict the rapid resource acquisition required by *F. bidentis*. Nevertheless, this interpretation requires experimental verification. By contrast, the lower invasion indices observed under low-dose SA-biochar with nitrogen should be interpreted cautiously. Because invasion indices are calculated from relative community composition, reductions in community invasibility or competitive advantage do not necessarily indicate suppression of the invader. Indeed, low-dose SA-biochar simultaneously promoted several growth traits of *F. bidentis*, suggesting that changes in invasion indices may partly reflect shifts in native species abundance or total community biomass rather than genuine reductions in invasion potential (Wang et al., 2020, 2021). Accordingly, invasion-control efficacy should be evaluated using both relative indices and absolute measures of invader performance, including biomass, abundance, and height, together with indicators of native community recovery (Zhang et al., 2024b; Dudenhöffer and Hulme, 2025; Li et al., 2026).

Nitrogen addition also reshaped community diversity and assembly, but again in a strongly dose- and feedstock-dependent manner. High-rate AP-biochar combined with nitrogen substantially increased Shannon and Simpson diversity (**Figure 3**), suggesting partial recovery of community structure following suppression of invasive plant dominance. Reduced competitive exclusion by *F. bidentis* may have increased resource availability and niche opportunities for subordinate native species, thereby improving community evenness (Li et al., 2025d; Zhang et al., 2025b; Sun et al., 2026; Zhang et al., 2026b). Nevertheless, several native growth traits also declined under this treatment. Thus, the increase in diversity should not be interpreted as complete restoration of native community function. Instead, it likely reflects reduced invader dominance and greater evenness in community abundance. Conversely, the decline in phylogenetic diversity under 1% AP-biochar combined with nitrogen suggests that low biochar application was insufficient to offset nitrogen-induced environmental filtering (Thomas et al., 2024; Wang et al., 2026a). Nitrogen enrichment may favor species with similar resource-acquisition strategies, thereby increasing phylogenetic clustering despite enhanced invasion resistance (Yin et al., 2024; Zhang et al., 2024a). Low-dose SA-biochar plus nitrogen showed the opposite pattern by increasing taxonomic and phylogenetic diversity while simultaneously promoting the growth of *F. bidentis*. Thus, greater biodiversity did not necessarily translate into greater invasion resistance. This finding reinforces the importance of distinguishing community diversity from community resistance when evaluating restoration outcomes.

Nitrogen co-addition further modified the effects of biochar on invasion by altering the performance-based niche of *F. bidentis* and phylogenetic turnover. Under AP-biochar, nitrogen consistently narrowed the niche breadth of *F. bidentis*, and modified βMPD, βMNTD, βNTI, and βNRI, particularly at higher application rates (**Figures 4**, **5**). However, because most βNTI and βNRI values remained within the ±2 threshold, these shifts provide limited evidence for strong deterministic environmental filtering. Instead, nitrogen appeared to modify phylogenetic turnover and species responses to AP-biochar, while stochastic processes likely remained important in community assembly. This interpretation is consistent with previous evidence that nitrogen enrichment and biochar-mediated changes in soil conditions can modify plant community structure (Khatri et al., 2024a; Thomas et al., 2024; Li et al., 2025d; Wang et al., 2026a). In contrast, phylogenetic responses to SA-biochar were weaker and more variable across application rates, paralleling its less consistent effects on plant performance and community resistance. Previous studies have linked biochar-induced rhizosphere changes and arbuscular mycorrhizal fungi with plant nutrient acquisition and competitive interactions (Zhang et al., 2017; Wang et al., 2025; Dong et al., 2026), although these mechanisms were not directly evaluated here.

Collectively, these findings indicate that nitrogen addition can redirect rather than simply mitigate the ecological effects of invasive plant-derived biochar. Our second hypothesis was therefore only partially supported. High-rate (5%) AP-biochar combined with nitrogen consistently constrained *F. bidentis*, strengthened community invasion resistance, and partially increased community diversity. In contrast, low- to moderate-rate (3% to 5%) SA-biochar with nitrogen frequently favored invader growth despite increasing some diversity metrics. The results further suggest that nitrogen-mediated changes in plant–microbe nutrient competition may complement biochar-driven nitrogen retention and nutrient stoichiometric shifts in explaining these contrasting responses, although this mechanism remains to be tested directly. Future assessments of invasive plant-derived biochar under increasing nitrogen deposition should therefore integrate invader performance, native recovery, microbial nutrient immobilization, resource partitioning, and community assembly rather than relying on a single invasion or diversity metric.

## 5 Conclusions

Our findings demonstrate that the ecological effects of invasive plant-derived biochar are strongly contingent upon biochar feedstock, biochar application rate, and nitrogen availability. Thus, our first hypothesis was only partially supported: heterospecific invasive plant-derived biochar did not consistently promote *Flaveria bidentis*, but certain treatments (3% AP-biochar and 5% SA-biochar) enhanced invasion primarily by suppressing native communities and weakening biotic resistance rather than by universally stimulating invader growth. Likewise, our second hypothesis was partially supported. Without nitrogen addition, 3% and 5% AP- and SA-biochar applications increased relative invasion risk by enhancing *F. bidentis’s* certain growth traits and reducing native community resistance. Nitrogen addition did not uniformly mitigate relative invasion; instead, it shifted biochar-mediated competitive interactions in a feedstock-dependent manner. 5% AP-biochar combined with nitrogen most effectively reduced invasive plant dominance, enhanced community resistance, promoted diversity recovery, and restricted the niche breadth of *F. bidentis*, whereas 1% and 3% SA-biochar with nitrogen frequently favored relative invasion. These results highlight that invasive plant-derived biochar should not be regarded as a universally beneficial restoration amendment, and its ecological risks must be evaluated before field application. Future studies should verify the underlying mechanisms by integrating long-term field experiments with measurements of soil nutrient cycling, rhizosphere microbial communities, particularly arbuscular mycorrhizal fungi, and plant–soil feedbacks under realistic nitrogen deposition scenarios to optimize biochar-based management of invaded coastal wetlands.

## Data Availability

The raw data supporting the conclusions of this article will be made available by the authors, without undue reservation.
