# Supplementary material for: Nitrogen addition determines whether invasive plant-derived biochar facilitates or suppresses Flaveria bidentis invasion
Source: Front Plant Sci. 2026 Oct 28;17:1942632. doi: 10.3389/fpls.2026.1942632 (PMC13752037; doi:10.3389/fpls.2026.1942632)
Supplement: Supplementary file 1 [file SupplementaryFile1.docx]

#### **Supplementary material for**

**Nitrogen addition determines whether invasive plant-derived biochar facilitates or suppresses Flaveria bidentis invasion**

Xin Pu^1,3,#^, Huijun Wu^1,#^, Jin Zheng^1,*^, Mengxuan He^2,*^, Jie Liu^1, 3^

^1^State Key Laboratory of Petroleum Pollution Control, China National Petroleum Corporation Research Institute of Safety & Environment Technology, Beijing 102206, China

^2^Faculty of Geography, Tianjin Key Laboratory of Water Resources and Environment, Tianjin Normal University, Tianjin 300387, China

^3^State Key Laboratory of Herbage Improvement and Grassland Agro-ecosystems; Center for Grassland Microbiome; College of Pastoral Agriculture Science and Technology; Lanzhou University, Lanzhou 730000, China

#These authors contributed equally to this work.

*Corresponding author:

Jin Zheng Email: zhengjin2810@163.com

Mengxuan He E-mail: hemengxuannku@126.com

**Text S1. Detailed calculation of nitrogen addition**

The amount of nitrogen added to each pot was calculated based on the target nitrogen deposition rate and the soil surface area of each pot.

(1) Soil surface area of each pot:

37 cm × 30 cm = 1110 cm² = 0.0000111 hm²

(2) Target nitrogen addition rate:

30 kg N ha⁻¹ yr⁻¹

(3) Total amount of nitrogen required per pot:

30 kg N ha⁻¹ × 0.0000111 ha = 0.000333 kg N = 0.333 g N

(4) Amount of nitrogen applied per application:

0.333 g N ÷ 2 = 0.1665 g N

(5) Amount of NH₄NO₃ required per application:

The molecular mass of NH₄NO₃ is 80 g mol⁻¹, and its nitrogen mass fraction is 28/80 = 0.35.

0.1665 g N ÷ 0.35 ≈ 0.476 g NH₄NO₃ ≈ 476 mg NH₄NO₃

**Table S1. The geographic coordinate of sampling sites.**

| **Sampling sites** | **Geographic coordinates** |
| --- | --- |
| Site 1 | 117°34′53″ E, 38°46′20″ N |
| Site 2 | 117°34′11″ E, 38°46′20″ N |
| Site 3 | 117°34′58″ E, 38°46′17″ N |
| Site 4 | 117°35′08″ E, 38°46′20″ N |
| Site 5 | 117°35′05″ E, 38°46′15″ N |
| Site 6 | 117°35′14″ E, 38°46′20″ N |
| Site 7 | 117°34′01″ E, 38°46′18″ N |
| Site 8 | 117°34′52″ E, 38°46′15″ N |
| Site 9 | 117°35′03″ E, 38°46′15″ N |
| Site 10 | 117°35′16″ E, 38°46′17″ N |

**Table S2. Physicochemical properties of experimental soil.**

| **Physicochemical properties** | **Value** |
| --- | --- |
| Soil salinity (g/kg) | 0.84 |
| pH | 8.54 |
| Soil water content (%) | 39.8 |
| Soil organic matter (g/kg) | 29.78 |
| Soil total nigrogen (g/kg) | 0.82 |
| Soil total phosphorus (g/kg) | 1.82 |
| Soil available potassium (mg/kg) | 281.68 |
| Soil available phosphorus (mg/kg) | 41.58 |
| Soil ammonium nitrogen (mg/kg) | 1.30 |

**Table S3. Physicochemical properties of two biochars.**

| **Properties** | ***S. alterniflora*** | ***A. palmeri*** |
| --- | --- | --- |
| pH | 9.8 | 10.7 |
| Carbon (%) | 35.81 | 41.88 |
| Hydrogen (%) | 4.55 | 2.08 |
| Nitrogen (%) | 0.67 | 0.68 |
| Sulfur (%) | 0.38 | 0.72 |
| H:C | 0.13 | 0.050 |
| C:N | 53.45 | 61.6 |
| Ash (%) | 11.2 | 11.5 |

**Table S4**. Experimental treatments. AP and SA denote biochar derived from Amaranthus palmeri and Spartina alterniflora, respectively. Biochar was applied at 1%, 3%, and 5% (w/w). Nitrogen (N) was added at 30 kg N ha⁻¹ yr⁻¹.

| **Treatments** | **Biochar feedstock** | **Biochar application**  **rate (%)** | **Nitrogen**  **(30 kg N hm^−^² yr^−^¹)** | **Description** |
| --- | --- | --- | --- | --- |
| Control | Without biochar | 0 | 0 | unamended control received neither biochar nor nitrogen |
| 1% AP-biochar | *A. palmeri* | 1 | 0 | 1% *A. palmeri* biochar without nitrogen addition |
| 1% AP-biochar with N | *A. palmeri* | 1 | 30 | 1% *A. palmeri* biochar with 30 kg N hm^−^² yr^−^¹ nitrogen addition |
| 3% AP-biochar | *A. palmeri* | 3 | 0 | 3% *A. palmeri* biochar without nitrogen addition |
| 3% AP-biochar with N | *A. palmeri* | 3 | 30 | 3% *A. palmeri* biochar with 30 kg N hm^−^² yr^−^¹ nitrogen addition |
| 5% AP-biochar | *A. palmeri* | 5 | 0 | 5% *A. palmeri* biochar without nitrogen addition |
| 5% AP-biochar with N | *A. palmeri* | 5 | 30 | 5% *A. palmeri* biochar with 30 kg N hm^−^² yr^−^¹ nitrogen addition |
| 1% SA-biochar | *S. alterniflora* | 1 | 0 | 1% *S. alterniflora* biochar without nitrogen addition |
| 1% SA-biochar with N | *S. alterniflora* | 1 | 30 | 1% *S. alterniflora* biochar with 30 kg N hm^−^² yr^−^¹ nitrogen addition |
| 3% SA-biochar | *S. alterniflora* | 3 | 0 | 3% *S. alterniflora* biochar without nitrogen addition |
| 3% SA-biochar with N | *S. alterniflora* | 3 | 30 | 3% *S. alterniflora* biochar with 30 kg N hm^−^² yr^−^¹ nitrogen addition |
| 5% SA-biochar | *S. alterniflora* | 5 | 0 | 5% *S. alterniflora* biochar without nitrogen addition |
| 5% SA-biochar with N | *S. alterniflora* | 5 | 30 | 5% *S. alterniflora* biochar with 30 kg N hm^−^² yr^−^¹ nitrogen addition |

**Table S5**. **The species composition of the plant communities emerging from the all treatment.**

| **Plant species** | **Family** |
| --- | --- |
| *Chenopodium album* | Amaranthaceae |
| *Chenopodium urbicum* | Amaranthaceae |
| *Kochia scoparia* | Amaranthaceae |
| *Suaeda glauca* | Amaranthaceae |
| *Artemisia lavandulifolia* | Asteraceae |
| *Bidens bipinnata* | Asteraceae |
| *Bidens biternata* | Asteraceae |
| *Erigeron canadensis* | Asteraceae |
| *Flaveria bidentis* | Asteraceae |
| *Ixeridium chinense* | Asteraceae |
| *Sonchus wightianus* | Asteraceae |
| *Trigonotis peduncularis* | Boraginaceae |
| *Humulus scandens* | Cannabaceae |
| *Calystegia hederacea* | Convolvulaceae |
| *Abutilon theophrasti* | Malvaceae |
| *Setaria viridis* | Poaceae |
| *Polygonum aviculare* | Polygonaceae |
| *Rumex patientia* | Polygonaceae |

**Table S6**. Three-way ANOVA of the effects of biochar feedstock, biochar application rate, nitrogen (N) addition, and their interactions on growth traits of invasive and native plants. Values represent F-values. Asterisks indicate statistical significance (**p* < 0.05; ***p* < 0.01; ****p* < 0.001; *****p* < 0.0001). DF, degrees of freedom.

| **Plant species type** | **Variable** | **Biochar feedstock**  **（DF:1）** | **Biochar application rate**  **（DF:2）** | **N addition**  **（DF:1）** | **Biochar feedstock×application rate**  **（DF:2）** | **Biochar feedstock×N addition**  **（DF:1）** | **Biochar application rate×N addition**  **（DF:2）** | **Biochar feedstock×application rate×N addition**  **（DF:2）** |
| --- | --- | --- | --- | --- | --- | --- | --- | --- |
| invasive | Aboveground biomass | **5.56 **** | **4.44 *** | **14.24 ***** | **8.81 **** | 2.98 | 0.23 | 0.09 |
| invasive | Abundance | 2.84 | 0.01 | **15.05 ***** | **3.96 *** | **4.30 *** | 3.13 | 0.26 |
| invasive | Height | **4.70 *** | 0.66 | 2.89 | **5.1 *** | **6.18 **** | **84.94 ****** | 0.01 |
| invasive | Diameter | 0.64 | 3.9 | 2.52 | 0.8 | 0.36 | **6.73 *** | 0.84 |
| native | Aboveground biomass | **61.75 ****** | **12.51 ***** | 0.95 | 1.55 | 2.55 | 0.23 | 0.07 |
| native | Abundance | **43.39 ****** | **80.73 ****** | **6.85 *** | **6.34 *** | **12.51 ****** | 1.10 | **14.53 ***** |
| native | Height | **64.34 ****** | 1.89 | **9.24 **** | 2.61 | **3.58 *** | 2.38 | 3.24 |
| native | Diameter | **54.33 ****** | 2.12 | **22.01 ****** | 0.96 | 1.84 | 0.70 | 1.45 |

**Table S7**. Three-way ANOVA of the effects of biochar feedstock, biochar application rate, nitrogen (N) addition, and their interactions on plant community diversity, invasion-related indices, ecological niche characteristics, and phylogenetic *β*-diversity. Values represent F-values. Asterisks indicate statistical significance (**p* < 0.05; ***p* < 0.01; ****p* < 0.001; *****p* < 0.0001). DF, degrees of freedom.

| **Variable** | **Biochar feedstock**  **（DF:1）** | **Biochar application rate**  **（DF:2）** | **N addition**  **（DF:1）** | **Biochar feedstock×application rate**  **（DF:2）** | **Biochar feedstock×N addition**  **（DF:1）** | **Biochar application rate×N addition**  **（DF:2）** | **Biochar feedstock×application rate×N addition**  **（DF:2）** |
| --- | --- | --- | --- | --- | --- | --- | --- |
| Shannon | **18.85 ****** | **18.79 ****** | **12.81 ***** | 3.36 | **6.59 **** | 3.78 | **7.18 **** |
| Simpson | **19.79 ****** | **27.45 ****** | **8.55 **** | 2.69 | **3.78 *** | 2.12 | **7.17 **** |
| Richness | **26.93 ****** | **21.45 ****** | 0.49 | **6.49 *** | 2.61 | 0.48 | **5.36 *** |
| PD | **22.75 ****** | **25.12 ****** | 0.06 | 3.80 | **3.29 *** | 0.16 | **11.95 ***** |
| Abundance | 0.83 | 2.14 | **13.34 ***** | 0.30 | **8.09 ***** | **5.70 *** | 0.71 |
| Height | **40.41 ****** | **5.41 *** | 1.72 | 0.15 | 0.88 | **26.08 ****** | 0 |
| Diameter | **18.25 ****** | 0.01 | 0.18 | 1.32 | 0.10 | 0.32 | 0.57 |
| Aboveground biomass | **56.19 ****** | **8.86 **** | 0.02 | 3.42 | **3.31 *** | 0.10 | 0.03 |
| CII | **26.64 ****** | **20.16 ****** | **6.08 *** | 2.60 | 2.44 | 0.9 | **5.87 *** |
| IRI | **12.02 ****** | **27.38 ****** | **8.24 **** | 0.81 | 0.29 | 0.03 | **11.85 ***** |
| CAI | **11.91 ****** | **27.42 ****** | **8.2 **** | 0.80 | 0.31 | 0.03 | **11.93 ***** |
| RII | **10.50 ****** | **9.2 **** | **17.44 ****** | **22.72 ****** | **5.04 *** | 1.42 | 0.15 |
| *β*MNTD | **7.05 ***** | **11.86 ***** | **4.08 *** | **62.61 ****** | **12.21 ****** | **34.33 ****** | 1.93 |
| *β*MPD | **152.82 ****** | **83.42 ****** | **73.7 ****** | **48.87 ****** | **16.1 ****** | **3.21 *** | **35.29 ****** |
| *β*NTI | 0.41 | 1.45 | 0.82 | 1.78 | 0.88 | 0.95 | 1.76 |
| *β*NRI | 0.17 | **4.09 *** | 1.35 | 1.86 | 0.52 | 2.08 | 1.21 |

**Table S8**. Adonis results for the effects of biochar feedstock, biochar application rate, nitrogen (N) addition, and their interactions on plant community composition. Asterisks indicate statistical significance (**p* < 0.05; ***p* < 0.01; ****p* < 0.001; *****p* < 0.0001)

| **Factors** | ***R^2^*** |
| --- | --- |
| Biochar feedstock | **0.0821 ***** |
| Biochar application rate | **0.0895 ***** |
| N addition | **0.0510 ***** |
| Biochar feedstock × Biochar application rate | **0.0361 **** |
| Biochar feedstock × N addition | **0.0738 ***** |
| Biochar application rate × N addition | **0.0375 **** |
| Biochar feedstock × Biochar application rate × N addition | 0.0031 |

**Fig. S1** Fourier-transform infrared spectra of *Amaranthus palmeri* and *Spartina alterniflora* biochar.
